# ICTV Virus Taxonomy Profile: *Nudiviridae*

**DOI:** 10.1099/jgv.0.001381

**Published:** 2020-01-14

**Authors:** Robert L. Harrison, Elisabeth A. Herniou, Annie Bézier, Johannes A. Jehle, John P. Burand, David A. Theilmann, Peter J. Krell, Monique M. van Oers, Madoka Nakai, ICTV Report Consortium

**Affiliations:** ^1^​ Invasive Insect Biocontrol and Behavior Laboratory, Beltsville Agricultural Research Center, USDA Agricultural Research Service, Beltsville, MD 20705, USA; ^2^​ Institut de Recherche sur la Biologie de l’Insecte, UMR 7261 CNRS/Université de Tours, Tours, 37200, France; ^3^​ Julius Kühn Institute, Federal Research Centre for Cultivated Plants, Institute for Biological Control, Darmstadt, 64287, Germany; ^4^​ Department of Microbiology, University of Massachusetts-Amherst, Amherst, MA 01003, USA; ^5^​ Summerland Research and Development Centre, Agriculture and Agri-Food Canada, Summerland, BC V0H 1Z0, Canada; ^6^​ Department of Molecular and Cellular Biology, University of Guelph, Guelph, Ontario N1G 2W1, Canada; ^7^​ Laboratory of Virology, Wageningen University, Wageningen 6708 PB, The Netherlands; ^8^​ Graduate School of Agriculture, Tokyo University of Agriculture and Technology, Tokyo 183-8509, Japan

**Keywords:** *Nudiviridae*, ICTV report, taxonomy

## Abstract

Members of the family *Nudiviridae* are large dsDNA viruses with distinctive rod-shaped nucleocapsids and circular genomes of 96–232 kbp. Nudiviruses have been identified from a diverse range of insects and crustaceans and are closely related to baculoviruses. This is a summary of the International Committee on Taxonomy of Viruses Report on the taxonomy of the family *Nudiviridae*, which is available at ictv.global/report/nudiviridae.

## Virion

**Fig. 1.**
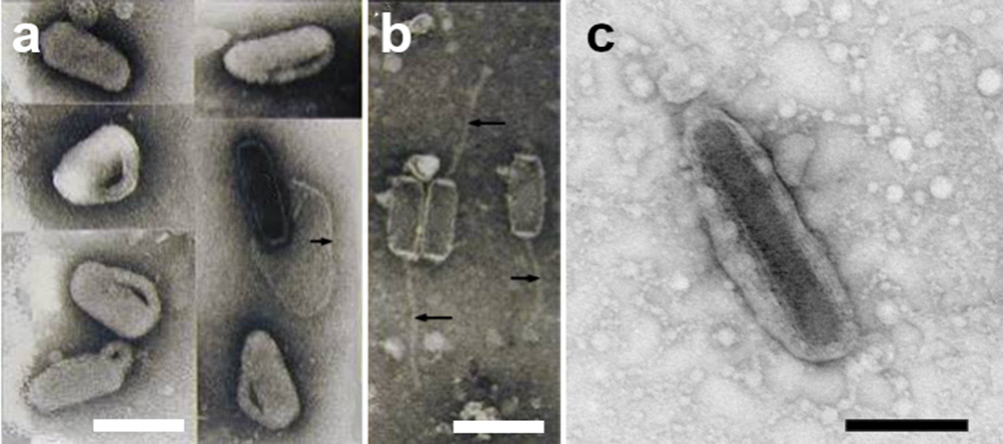
Negatively-stained virions (a) and nucleocapsids (b) of Oryctes rhinoceros nudivirus (genus *Alphanudivirus*). Arrows indicate (a) the membrane from a disrupted virion, (b) tail-like appendages. (c) A virion of Helicoverpa zea nudivirus 2. Bars, 0.2 µm. Micrographs (a) and (b) modified and reprinted from [5] with permission from Elsevier, (c) produced by Jean Adams (USDA-ARS).

Nudivirus virions consist of cylindrical nucleocapsids enveloped to produce ellipsoidal or rod-shaped virions of variable length and width (Fig. 1, Table 1). In some cases, virions are assembled into occlusion bodies, with a matrix consisting of a protein similar to the baculovirus polyhedrin protein [1] or of a completely unrelated protein [2].

**Table 1.** Characteristics of members of the family *Nudiviridae*

Typical member:	Oryctes rhinoceros nudivirus Ma07 (EU747721), species *Oryctes rhinoceros nudivirus*, genus *Alphanudivirus*
Virion	Enveloped, rod-shaped or ellipsoidal, compact (approximately 100×200 nm) or elongated (approximately 81×415 nm)
Genome	A single covalently closed circular dsDNA molecule of 96–232 kbp encoding 89–155 proteins
Replication	Nuclear, including nucleocapsid assembly and envelopment
Translation	From mRNAs transcribed from viral DNA
Host range	Immature and adult stages of insects and crustaceans
Taxonomy	Multiple genera and species

## Genome

**Fig. 2.**
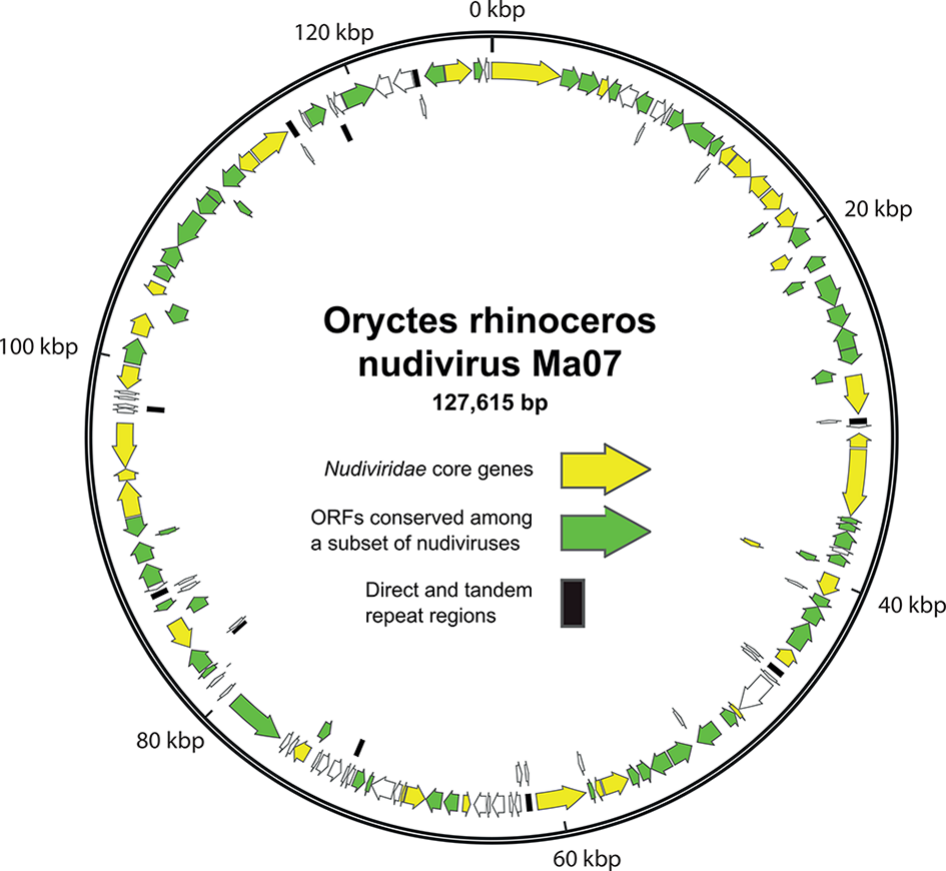
Genome map of Oryctes rhinoceros nudivirus isolate Ma07. Positions and orientations of annotated ORFs are represented as arrows, with filled arrows corresponding to core genes (yellow) and ORFs conserved among other nudiviruses (green). Locations of direct (dr) and tandem (tr) repeat sequence regions are also shown.

The nudivirus genome is a single, covalently closed circular molecule of dsDNA, with approximately 89–155 potential protein-encoding ORFs (Fig. 2). These ORFs are distributed throughout the genome in either orientation. ORF content and order can vary significantly in members of different species. Most genomes also contain regions of short repeated sequences. The 32 ORFs present in all nudivirus genomes make up the set of core genes [1], many of which are homologues of baculovirus core genes [3]. These core genes include ORFs encoding DNA polymerase B and baculovirus RNA polymerase subunit homologues that probably participate in viral genome replication and gene expression, respectively.

## Replication

After virus entry, replication occurs in the nucleus and is accompanied by nuclear hypertrophy. Progeny nucleocapsids are assembled and enveloped within the nucleus. Mature virions either bud from the cytoplasmic membrane of infected cells or are released upon cell lysis. A persistent, asymptomatic infection may be established.

## Taxonomy

*Alphanudivirus*. Members of two species in this genus include Oryctes rhinoceros nudivirus from the coconut rhinoceros beetle, *Oryctes rhinoceros*, and Gryllus bimaculatus nudivirus from a field cricket, *Gryllus bimaculatus*. Virions of these viruses are relatively compact, although a tail-like appendage extends from Oryctes rhinoceros nudivirus nucleocapsids (Fig. 1a, b).

While Oryctes rhinoceros nudivirus exclusively infects adults and larvae of *O. rhinoceros*, Gryllus bimaculatus nudivirus can infect different species of field crickets [4]. Infections can result in death of the host. Oryctes rhinoceros nudivirus has been developed and applied successfully to control infestations of *O. rhinoceros* on coconut palm trees [5].

*Betanudivirus*. The species *Heliothis zea nudivirus* is represented by two isolates, Heliothis zea nudivirus 1 and Helicoverpa zea nudivirus 2, whose genome sequences are 93.5 % identical. The virions of these viruses are longer and thinner than those of the alphanudiviruses (Fig. 1c and Table 1).

Heliothis zea nudivirus 1 initially causes a lytic infection, but surviving cells harbour the virus in a persistent state facilitated by virally encoded micro-RNAs [6]. Although Heliothis zea nudivirus 1 can infect cell lines from the noctuid *Helicoverpa* (formerly *Heliothis*) *zea* and other host species, attempts to infect *H. zea* insects have been unsuccessful. Helicoverpa zea nudivirus 2 can infect *H. zea* larvae, but replication is constrained to reproductive tissue and may result in gonadal atrophy and sterility. Helicoverpa zea nudivirus 2 is transmitted both vertically to progeny and between adult moths during mating [7].

## Resources

Current ICTV Report on the family *Nudiviridae*: ictv.global/report/nudiviridae.
